# Public advocates, private advisors: the autonomy, function, and influence of the President's Council of Advisors on Science and Technology

**DOI:** 10.3389/frma.2024.1455510

**Published:** 2024-12-24

**Authors:** Kenneth M. Evans, Kirstin R. W. Matthews

**Affiliations:** ^1^Science and Technology Policy Program, Baker Institute for Public Policy, Rice University, Houston, TX, United States; ^2^Rice Innovation, Rice University, Houston, TX, United States

**Keywords:** expertise, science-policy nexus, advisory committees, public policymaking, negotiation

## Abstract

US national expert advisory bodies related to science, technology, and innovation (STI) policy have a wide range of missions, governing structures, operational practices, cultures, and impact on federal policymaking. This paper offers an analytical framework for assessing the autonomy, function, and influence of the President's Council of Advisors on Science and Technology (PCAST), a federal advisory committee consisting of 30 elite scientists, engineers, and industry leaders appointed by and advising the president. We demonstrate that PCAST carries both a strong instrumental advisory role, providing substantive advice to White House STI policy development, and a significant symbolic advisory role, offering visible public support to presidential decisions and initiatives related to STI. However, we find that the council's engagement with either or both roles has shifted depending on its available resources, the policy agenda of the administration it serves, the level of presidential attention, and the priorities of council leadership. The paper concludes with recommendations to guide future PCASTs in fulfilling their mission and appropriately influencing US national STI policy.

## 1 Introduction

Independent, high-level science advice to governments has never been more salient to policymaking, nor more publicly debated (Eyal, 2019). The US science, technology, and innovation (STI) advisory system consists of a large, decentralized network of government, government-sponsored, and independent sources of advice with varying levels of autonomy, prestige, and represented interests (Holland and Lane, 2018). The vast majority of formal STI policy advice solicited by the federal government occurs within regulatory and grantmaking federal STI agencies focused on specific areas of scientific research and development (R&D) and technology programs (Ginsberg and Burgat, 2016a; Stine, 2005). These committees help inform rulemaking, set agency priorities, conduct peer review of competitive grants, and evaluate STI programs. These tasks are typically performed by working scientists or industry representatives with little to no formal training in policy (Cozzens, 2009). Most federally-sponsored STI advisory committees fall under the Federal Advisory Committee Act (FACA), which governs committee operations, membership balance, and public transparency (Ginsberg and Burgat, 2016b; P. L. 92-463, 1972). Such committees have been the subject of extensive scholarship on the politics of expertise within the US STI policymaking system (Brown, 2008; Campbell, 1998; Fleisher, 2015; Jasanoff, 1990; Moffitt, 2010; Pielke, 2007).

Only five expert committees are tasked with making recommendations for improving the overall US national STI R&D enterprise: the Defense Science Board (DSB), JASON, the National Academies of Sciences, Engineering, and Medicine (NASEM), the National Science Board (NSB), and the President's Council of Advisors on Science and Technology (PCAST).^1^ There is a comparative dearth of academic literature or policy scholarship on these committees, which operate under various legal conditions (Blair, 2016; Finkbeiner, 2006; Evans and Matthews, 2018, 2024; Hart, 2014; Holland and Lane, 2018; Smith, 1992). In particular, there remains strikingly little published research on PCAST, especially considering the historical importance of its Cold War analog, the President's Science Advisory Committee (PSAC) (Golden, 1993; Herken and Leone, 2000; Wang, 2008). Even among recent scholarship focused on the history of federal STI advising, PCAST is rarely discussed by historians, former government officials, or policy scholars (Blanpied, 2010; Holland and Lane, 2018; Lubell, 2019; Pielke and Klein, 2010). This paper offers a comprehensive analysis of PCAST's organization, operations, and policy activities from its creation by President George H.W. Bush through the Biden administration.

PCAST is a FACA committee appointed by and advising the president that consists of roughly 30 elite scientists, engineers, academic leaders, and senior industry executives. The committee is managed by the White House Office of Science and Technology Policy (OSTP) and chaired or co-chaired by the director of OSTP, who is informally called the president's “science advisor.”^2^ PCAST was created by President George H.W. Bush in 1990. However, the PCAST advisory mechanism—a standing committee of independent experts reporting to the president—dates back to the Truman administration and the creation of the Scientific Advisory Committee (SAC) in 1951 within the Office of Defense Mobilization (ODM). ODM-SAC was tasked with providing “independent advice on scientific matters especially as [it] regards the objectives and interrelations of the several federal agencies engaged in research of defense significance” (Truman, 1951).

While its name and stature within the White House have changed significantly over time, every president since President Truman has employed some form of the PCAST mechanism to advise on the wide range of policy issues that rely on STI expertise (Evans and Matthews, 2018, 2024). In its 2021 charter, issued through executive order by President Biden, PCAST advises the president “on matters involving policy affecting science, technology, and innovation, as well as on matters involving scientific and technological information that is needed to inform public policy relating to the economy, worker empowerment, education, energy, the environment, public health, national and homeland security, racial equity, and other topics” (Executive Office of the President, 2021). This broad mission encompassing nearly every facet of domestic policy reflects a shift in the use of STI advisors by policymakers in the decades following WWII. Rather than just “speaking truth to power” on areas that require specialized technical knowledge, expert committees are also asked to provide judgement on broader social and political issues related to STI (Jasanoff, 2005; Maasen and Weingart, 2005; Eyal and Medvetz, 2023).

This paper assesses PCAST's advisory role, as defined by Dluhy (1981) as “the set of prescribed behaviors and relationships that are in accordance with the expectations that other have toward that role and any incumbent of that role.” Tracing the council's charter language, membership, meetings, policy products, and leadership structure from 1990 to 2023, we discuss the expectations set for PCAST by its appointing authority; how PCAST has met those expectations; and in turn, how PCAST has been received by policymakers. We conclude with recommendations to help ensure future PCASTs fulfill their stated mission and position themselves to appropriately influence White House decision-making related to STI.

## 2 The political uses of scientific expertise

STI policy development and implementation involve a wide range of actors, interests, and evidence, each of which are difficult to track from ideation to tangible societal outcomes (Christensen, 2023; Freeman and Maybin, 2011; Haas, 2004; Head, 2008). On their surface, STI advisory committees participate in the policy process by providing outside expertise to their appointing authority based on public or private deliberations between committee members (Krick, 2015). In practice, advisory committees can serve multiple functions beyond offering guidance, insights, or opinions on policy. STI committees can provide a cost-effective means of expanding the expertise or institutional capacity of government offices or agencies, develop policy alternatives to existing proposals, help in crisis response management, and reduce the workload of government staff (Bybee, 1990; Campbell, 1998; Stine, 2005). Committees can also be used as political tools by public officials to legitimize existing policy proposals, demonstrate competency, serve as scapegoats for unpopular policy decisions or inaction, and tackle intractable or politically controversial topics (Bybee, 1990; Feinstein and Hemel, 2019; Zegart, 2004).

Krick (2015) categorizes these roles into two groups: the instrumental dimension of policymaking and the symbolic use of expertise. The instrumental role refers to the committee's primary function to offer policy recommendations or options on complex topics, increase the appointing authority's understanding of an issue, and improve government performance in a specific policy arena. Solutions to policy challenges arrive from committee negotiations, whereby the represented interests and perspectives within the committee are reconciled. This role also contains several potential subfunctions that use the private networks, resources, and eminence of expert committee members. These subfunctions include the potential for increased uptake of committee recommendations and a wider dissemination of public resources through broad stakeholder engagement. In this way, STI advisory committees are positioned to influence government decision-making by providing a forum for dialog between experts and the public (Brown, 2009; Collins et al., 2023).

The symbolic role of STI advisory committee leverages the represented expertise and prestige of committee members to support, justify, or legitimize government decisions (Boswell, 2008; Krick, 2015). This function can be extended to predetermined positions on policy issues or the postponement of decision-making (Zegart, 2004). In this role, the committee is used as a political instrument in service to the needs and agenda of the administration or as a method to respond to Congressional or public calls for action.

Committees can and often do serve both functions simultaneously. However, for instrumental use, overt or *de facto* government control of committee operations and deliberations could undermine the public perception or credibility of committee recommendations. In contrast, for symbolic use, direct government involvement is appropriate to ensure that committee advice is relevant and actionable to policymakers (Krick, 2015; Verhoest et al., 2004). This inherent conflict of the political uses of STI expertise is what Bressers et al. (2018) describes as the “contested autonomy” of expert advisory bodies: the tradeoffs in committee organization and operations that facilitate or impede the committee's ability to meet the needs of its appointing authority while still being viewed as independent by the public.

## 3 Analytical framework and methodology

Despite the ample scholarship dedicated toward national STI advisory systems, measuring expert influence is a challenging and evolving field of research (Christensen, 2023). Further, although many areas of policy development have been “scientized,” STI policy remains largely unscientific (Stucke, 2011; Maasen and Weingart, 2005). There remains limited scholarship that assesses the policy impact of national STI expert bodies, much of which has been focused outside of the United States (Cambrosio et al., 1990; Christensen and Holst, 2017; Craft and Halligan, 2015; Jasanoff, 2013; Krick, 2018; Li, 2021). This study builds on complementary analytical frameworks developed by Halligan (1995), Bressers et al. (2018), Craft and Howlett (2012), and Krick (2015) to assess the instrumental and symbolic uses of PCAST in presidential STI policymaking. The analysis also draws from conceptual methods for evaluating the role of individual science advisors or STI advisory committees in the policy process (Dluhy, 1981; Gluckman et al., 2021; Pielke, 2007). This scholarship offers language for describing how STI advisers navigate the blurred lines between facts and values necessary to serve as “knowledge brokers” by translating scientific data and analysis to policymakers. This paper uses PCAST as a case study of a “boundary organization” that operates at the science-policy interface and serves as a forum for engagement between scientists and government officials (Boswell, 2018; Guston, 1996; Hoppe, 2010; Wesselink and Hoppe, 2020).

We use four criteria for assessing PCAST's instrumental and symbolic advisory roles:

(1) The formal or *de facto* independence of committee operations, i.e., the legal, organizational, and economic characteristics that enable or limit its advisory roles (Bressers et al., 2018; Craft and Howlett, 2012; Halligan, 1995).
(2) The decision-making conditions of committee activities, i.e., its size, membership demographics, division of labor, openness of committee negotiations, breadth of stakeholder engagement, and available resources (Bressers et al., 2018; Krick, 2015).
(3) The types of advice presented to stakeholders, i.e., whether its advice is reactive or anticipatory; the intended audiences for its advice; the substantiveness of its policy recommendations; and the format and content of advisory activities (Bressers et al., 2018; Craft and Howlett, 2012).
(4) The degree of publicity of committee operations and products, i.e., the attention paid toward creating a positive public image of the committee and the commitment of the committee and its membership to media engagement about its policy activities (Boswell, 2008; Krick, 2015).

Greater formal or *de facto* independence, private and autonomous deliberations, substantive policy contributions, and lower publicity signal a more prominent instrumental advisory role. A visible dependency of committee operations on its appointing authority, controlled negotiations, and highly positive public image indicates a more symbolic advisory role. Using this framework, we demonstrate that PCAST has carried strong instrumental and symbolic roles, but that its engagement with one or both advisory functions has changed across presidencies and council leadership.

Our analysis draws from three sources of data: 19 oral history interviews with former members of PCAST, official records and published materials from PCAST, OSTP, and other government agencies and offices, and archival records related to PCAST operations and its membership from 1989 to 2023. The oral history interviews, conducted by the authors and collaborators between 2019 and 2023, were sampled to be inclusive of presidential administrations from President George H.W. Bush through President Trump, and representative of the racial, ethnic, gender, and geographic demographics of PCAST's membership (Science History Institute, 2024). The interview data were used to understand how PCAST members viewed their own involvement with the council, their experiences engaging with senior White House officials, and their perspectives of the autonomy and policy influence of council activities.

The interview data were complemented by a review of news media and published PCAST documents, such as policy reports, committee charters, and notices of PCAST plenary meetings. Additionally, the analysis relies on a close reading of textual records housed in the White House Scientist and Science Policy Dynamic Digital Archive, such as personal correspondence, internal White House communications, and draft policy reports and memoranda (Woodson Research Center, 2024). These documents were examined for historical background into PCAST operations, its intended use and reception by policymakers, and the public perception of PCAST activities of journalists and other close observers of US national STI policy.

## 4 Exploring PCAST's advisory roles across time

### 4.1 Formal or *de facto* independence

We assess PCAST's independence by examining its legal, organization, and economic autonomy from government control. Legal autonomy refers to the statutory boundaries that govern committee operations. Organizational autonomy relates to the characteristics, perspectives, and backgrounds of the council membership and staff. And economic autonomy describes the source of the council's budget and input on planning council activities and products (Bressers et al., 2018; Craft and Howlett, 2012; Halligan, 1995).

#### 4.1.1 Legal autonomy

PCAST is a temporary advisory committee established by the president through executive order and renewed every 2 years. It is designated as a national policy issue advisory board under FACA, which mandates certain membership balance, transparency, and reporting requirements (Bybee, 1994). FACA requires that committee membership be “fairly balanced in terms of the points of view represented and the functions to be performed.” This clause works to ensure committee activities and policy recommendations are “not inappropriately influenced by the appointing authority or by any special interest” (P. L. 92-463, 1972). Membership balance is evaluated in the context of the PCAST's mission to inform White House STI policy, and the “geographic, ethnic, social, economic, or scientific impact” of its recommendations (US General Services Administration, 2011). In practice, FACA allows the appointing authority—in this case, the president—significant freedom to determine appropriate committee balance, presenting an opportunity to design the committee to align with administration needs or its broader political agenda (Brown, 2008). FACA requires its managing agency, OSTP, to report annually to the General Services Administration on the committee's membership balance plan, activities, and actions in response to its policy recommendations (US General Services Administration, 2024).

FACA also mandates that PCAST meetings must be publicly announced on the Federal Register at least 15 calendar days in advance of the meeting and that meetings and meeting materials be open to the public unless otherwise justified by the president or an affiliated agency head for national security reasons or other privacy concerns. However, PCAST's large network of working groups and subcommittees, where much of the report development and internal deliberations take place, are not subject to the same oversight. These groups report to OSTP, not to the General Service Administration (GSA), and their meeting materials are not published. Further, most PCAST materials—e.g., draft policy proposals, communications between committee members, internal White House planning documents—are considered “predecisional” under Freedom of Information Act (FOIA) and restricted from public viewing (P. L. 89-487, 1967). Only published consensus reports, final meeting agendas, and meeting minutes approved by OSTP are published in accordance with FACA requirements. Additionally, FACA's transparency requirement, with advanced planning, is straightforward to navigate; if negotiations require privacy, the science advisor, in their capacity as OSTP Director, can schedule a closed-door meeting or portion of the meeting, providing a forum for candid discussion.

Indeed, when PCAST was first established under President George H.W. Bush, the question of whether the science advisor could legally close a FACA meeting was brought before the White House counsel. The counsel's office found “no disqualifying conflict” between the science advisor's “duties as head of OSTP and chairman of PCAST,” and that “he may close all of some portion of PCAST's meetings in the ordinary course of his duties as head of OSTP” (Bybee, 1990). This interpretation of the authority of the science advisor was directly challenged just 2 years later when OSTP was sued by a group of publishers for not meeting FACA's transparency requirements (Reppert, 1992). The lawsuit forced the release of all previously unreleased PCAST materials, such as meeting agendas and minutes, and prompted the publication of all seven of the George H.W. Bush administration's PCAST reports *en masse* in December 1992—just 1 month before the end of President Bush's term. The lawsuit likely influenced the PCAST operations in the Clinton and George W. Bush administrations. PCAST held 11 closed-door plenary meetings in 3 years under President George H.W. Bush and only one during the 16 years that followed (Federal Register, 2024). President Obama reversed the trend of fully public PCAST plenary meetings; all 46 council meetings scheduled during his presidency included a closed-door portion, which he reportedly attended with some regularity (Holdren, 2023; Press, 2022, 2023; Savitz, 2021).

While FACA forbids federal officials from interfering with committee findings or published policy recommendations, in practice PCAST often serves as a public-facing extension of the White House policymaking apparatus. Schaal (2022), who served on President Obama's PCAST described the council as a “reflection of the executive branch.” Unlike NASEM, which maintains formal independence from government officials, PCAST is able to “socialize” its recommendations before they are issued (Press, 2022, 2023; Schaal, 2022). For example, D. Allan Bromley, chairman of PCAST under President George H.W. Bush, circulated PCAST's reports to Cabinet members to solicit feedback before finalizing and publishing recommendations (PCAST, 1992a).

In short, with the exception of its public plenary meetings, PCAST operates in private in accordance with FACA and FOIA, laws which, ironically, were put in place to increase public transparency. However, the privacy granted by these statutes affords PCAST greater operational autonomy from government control and signals a strong instrumental advisory role. This autonomy, by facilitating more candid debate among its members, allows for more effective internal deliberation that could contribute to the quality of its recommendations.

#### 4.1.2 Organizational autonomy

PCAST is chaired or co-chaired by the president's science advisor, an informal title that refers to either or both of the positions assistant to the president for science and technology (APST) or director of OSTP. As APST, the science advisor is a senior staff member of the Executive Office of the President (EOP) and a confidential adviser to the president holding executive privilege. As OSTP Director, the science advisor is the Senate-confirmed head of a statutory agency and subject to congressional oversight, as described in OSTP's founding legislation (P. L. 94-282, 1976).^3^ The dual-hatted role of the science advisor is uncommon among presidential appointees: assistants to the president are typically not confirmed by the Senate and few Senate-confirmed directors of an office in the EOP are designated as assistants the president. The science advisor serves as PCAST chair or co-chair and the council's only federal employee. If the science advisor holds both titles of APST and OSTP Director, they serve as PCAST co-chair in their capacity as APST, rather than as OSTP Director, suggesting PCAST may have more autonomy from congressional oversight.^4^ This arrangement also allows the science advisor, as OSTP Director, to close PCAST meetings under FACA. PCAST members are not often called before Congress to testify and the science advisor, when called to testify, does so in their capacity as OSTP Director.

PCAST's 2021 charter under President Biden also allowed for up to two external, nonfederal co-chairs. However, in two administrations—Presidents George H.W. Bush and Trump—the science advisor served as the sole chair. This flexibility affords the White House significant control in institutionalizing PCAST's independence through the design of the council's leadership structure; the science advisor serving as sole chair suggests less organizational autonomy.^5^ Moreover, a federal member serving in a leadership position on a FACA committee is uncommon. In PCAST's case, the science advisor holding or sharing the chairpersonship is likely due to the historical precedent of PSAC rather than common FACA practices. Federal officials are more commonly included on FACA committees as *ex-officio*, non-voting members, signaling greater independence from their appointing authority (Sargent and Shea, 2020). In contrast, the science advisor's position as chair or co-chair of PCAST suggests the council is dependent on the government for direction, priority setting, and consensus building.

Presidential involvement, especially for establishing prioritized areas for study early in the administration, indicates an increased level of government control of PCAST. While the participation of the president has varied, each president, with the exception of President Trump, met with PCAST toward the start of the administration and periodically throughout their term. President George H.W. Bush famously held PCAST's inaugural meeting at Camp David, an occurrence that only happened once more under President Obama (Savitz, 2021). President Clinton was criticized for not meeting with his PCAST until its third meeting and rarely met with the group in the 8 years that followed (Goodwin, 1995; Lawler, 1997; Wu, 2001).^6^ President George W. Bush's PCAST was seen by STI policy advocates as inactive (Kelly et al., 2004). However, other accounts describe semi-regular and direction engagement between President Bush and PCAST (Dicciani, 2022; Kvamme, 2011; Marburger and Kvamme, 2008; Proenza, 2023). President Obama's PCAST saw increased direct involvement with the president, as well as regular, closed-door meetings and briefings with senior members of the Obama administration (Moniz, 2020; Press, 2022, 2023; Savitz, 2021). While President Trump never met with his PCAST, President Biden resumed occasional, direct presidential participation in PCAST meetings during his presidency. President Biden met with the council once or twice per year, a frequency similar to Presidents George H.W. Bush, Clinton, and George W. Bush (White House, 2023a).

#### 4.1.3 Economic autonomy

PCAST is funded entirely by the federal government, with rare exceptions to cover report costs during times of budget austerity. The budget, provided by either OSTP or the Department of Energy, covers staff support, travel costs for plenary meetings, and report development. Advisory committees fully financed by their appointing authority are subject to a high level of government control, signaling less independence and a stronger symbolic role. However, Bressers et al. (2018) argues that “from an economic perspective, government budgets for policy advisory bodies promote autonomy because it prevents them from having to gather private funds from actors with special, strategic interests in the advice provided to government.” PCAST's charter, as discussed in the following sections, allows for both solicited and unsolicited, but government approved, advice.

### 4.2 Decision-making conditions

Decision-making conditions refer to committee size and professional demographics of its membership; the division committee responsibilities and relative authority of federal and nonfederal members; and the available financial and personnel resources (Bressers et al., 2018; Krick, 2015). Krick (2015) describes the ideal “institutional conditions of real interaction and successful conflict resolution” to include “a lack of time constraints, open and genuine interaction, a limited number of participants, opacity of the interaction process, and mediation mechanisms.” PCAST has approached these conditions differently across time, presidential administrations, and committee leadership. PCAST, as described above, has the flexibility to ensure private, substantive deliberations between committee members and between committee members and senior government officials, signaling a strong instrumental advisory role.

#### 4.2.1 Available resources

PCAST is managed by OSTP but funded the Department of Energy, an unusual arrangement stemming from a lack of operating funds to support council operations under President Obama (Blevins, 2023). In fiscal year 2012, OSTP's budget was cut by one-third ($2.1 million) after congressional leaders condemned the office for bilateral dialogue with Chinese counterparts in violation of the so-called Wolf Amendment, a rider to annual appropriations that forbids such activities in OSTP and NASA (Ronci, 2019). Prior to this change, PCAST was funded by OSTP, with an annual budget of between $200,000 and 1.4 million and an average of roughly $600,000 (Evans and Matthews, 2018; US General Services Administration, 2024). While unusual, the change allowed President Obama to fund PCAST at levels consistently higher than the average budget in prior and subsequent administrations.

Even before the cuts to OSTP's budget under President Obama, PCAST struggled to find sufficient funding for its operations during the George H.W. Bush and Clinton administrations (Carnegie Commission on Science, 1997). Bromley encountered significant pushback from EOP staff related to OSTP activities, including PCAST. William Wells Jr., who served as OSTP's chief of staff under President George H.W. Bush, chronicled PCAST's financial difficulties in a letter to President Clinton's first science advisor, John H. “Jack” Gibbons. Wells Jr. wrote that “when it became clear that a budget increase was necessary to fund PCAST operations in 1991, [White House Office of Management and Budget (OMB) Director Richard G.] Darman and (OMB Associate Director Robert E.) Grady required Dr. Bromley to tell Senator (Barbara A.) Mikulski that it had to come out of ‘Mission to Planet Earth' [a major Bush administration space policy initiative] knowing that it would embarrass him” (Wells, 1993).^7^ Toward the end of President Clinton's second term, Neal Lane, the second science advisor to President Clinton, and John Young, PCAST's external co-chair, sought to reduce the number of plenary PCAST meetings to just twice a year due to lack of available resources (Porter, 1998). President Clinton's PCAST was so short on resources, one PCAST member, David E. Shaw, went as far as to fund a report on education technologies that he chaired using his own private money, which became known as *The Shaw Report* (Malcom, 2020; PCAST, 1997). Additionally, two late-term Clinton era reports on ecological resources and biodiversity were funded largely by outside organizations (PCAST, 1998, 2001). While these reports are exceptions to PCAST's budgeting structure, they indicate the members can self-fund or seek outside funding to advance policy interests PCAST believes to be worthy of presidential attention.

Budgets for PCAST recovered under President's George W. Bush, and later during the Obama administration, at levels that continued through the Trump and Biden administrations (Evans and Matthews, 2018). However, PCAST's budget still remains comparatively modest to the operational costs of other significant US national STI advisory bodies: NSB and DSB have annual operating budgets of roughly $5 million, just short of OSTP entire annual appropriations from Congress. By comparison, PSAC's annual budget, adjusted for inflation, totaled over $10 million (Beckler, 1974). Insufficient resources hamper PCAST's ability to fulfill its instrumental role. Adequate staff, funding, and regular plenary meetings facilitate ideal deliberative conditions among PCAST members, policymakers, and other stakeholder groups inside and outside of government.

#### 4.2.2 Committee size and represented perspectives

In its most recent charter issued by President Biden, PCAST allowed for up to 32 members. Members are described as “distinguished individuals and representatives from sectors outside of the federal government … [with] diverse perspectives and expertise in science, technology, and innovation” (Executive Office of the President, 2021). With the exception of the science advisor, all of PCAST's members are from the private sector and hired as special government employees (SGEs). SGEs are temporary government employees selected for their specific expertise who retain their professional affiliations during their service on PCAST.

Members are formally appointed by the president and announced alongside regular government employees (RGE). In practice, the science advisor, in coordination with the White House chief of staff, is responsible for identifying and winnowing the list of potential candidates before it is approved by the president. PCAST members serve at the pleasure at the president, indicating that the president can ask them to step down at any point during their service. Only 12 PCAST members have stepped down mid-term since PCAST was created in 1990 through the Trump PCAST. While it is challenging to identify the cause of every departure, most members who left mid-term did so to take senior positions within the government or appointments in other high-level FACA committees.^8^

The size of PCAST has shifted across presidential administrations, ranging from just 14 members during President Trump's first term to 35 members under President George W. Bush. Overall, PCAST's average number of members across administrations from 1990 to 2023 was 24 (Evans and Matthews, 2024). A larger membership can inhibit the effectiveness of the committee, making consensus building and management more challenging for PCAST leadership. For example, in a letter to the successors of PCAST, PCAST co-chairs under President George W. Bush John H. Marburger III and E. Floyd Kvamme (2008) stated that 35 members was too large to be managed effectively and that a quarter of the council's membership became inactive with time. A range of 20–30 principals allows for healthy deliberations without limiting the range of represented perspectives of membership (Krick, 2015; Raiffa, 2007).

FACA's balance requirement mandates the president build a diverse roster of PCAST members. However, FACA's language offers the administration wide authority to interpret how to ensure balance of represented perspectives, which can include their scientific expertise, career background, geographic location, and gender, race, and ethnicity (Brown, 2008; US General Services Administration, 2011). Each administration has approached the balance requirement differently, leading some administrations to focus more on professional perspectives (i.e., the representation of scientific disciplines and career experience) and others on social perspectives (i.e., the inclusion of social groups and geographic locations). PCAST's membership consists of elite scientists and engineers, including Nobel Prize winners and members of NASEM; academic leaders; senior executives of major technology and defense companies; and other highly visible and respected research professionals, such as astronauts and former Cabinet-level government officials. Most PCAST members were from academia; the remaining members were roughly split between two-thirds with career experience in private industry and one-third with experience in government service. President George W. Bush's PCAST was the exception; it consisted of a strong majority of members with career backgrounds in industry.^9^ PCAST membership has been overwhelmingly white and male, although representation of women and minoritized and historically marginalized racial and ethnic groups on PCAST has increased modestly with time (Evans and Matthews, 2024). President Biden's PCAST was an outlier, with an atypically diverse membership. Women made up half of the Biden administration's PCAST (sixteen of thirty-two members), and non-white members made up roughly one-third (eleven members).

As SGEs, PCAST members are subject to less restrictive conflict-of-interest (COI) regulations than RGEs. Each member is issued a waiver for financial conflicts, justified by “the need for their services [outweighing] the potential for a conflict of interest posed by the financial interest involved” (US Office of Government Ethics, 2021). In some administrations, notably under President Obama, PCAST members were also given security clearances to discuss classified matters, and some closed-door meetings are held in sensitive compartmented information facility (SKIFs), secure facilities within the White House or elsewhere (Gates, 2021; Sargent and Shea, 2020). The COI waivers, appropriate issuance of security clearances, and flexibility of open vs. closed meetings facilitates more candid discussion between members, offering a means for improving PCAST's deliberative capacity and the quality of its policy recommendations.

#### 4.2.3 Division of committee responsibilities

Plenary meeting scheduling, agenda-setting, working group or subcommittee assignments, and moderating of plenary sessions are performed by PCAST co-chairs. Both public and private meetings are typically attended by members of OSTP staff and representatives from federal STI agencies and Cabinet departments, as well as other public and private stakeholder groups. These individuals are often asked to make subject matter presentations to help inform PCAST's activities.

PCAST's charter affords significant flexibility in the council's division of responsibilities, and the ability for PCAST members to raise issues for possible study and volunteer to chair such activities. However, final decisions for areas of focus must are approved by the science advisor in consultation with the president, the White House chief of staff, and other senior staff members of the EOP. A close working relationship between the science advisor and PCAST encourages candor among members and can increase the uptake and representation of independent views in PCAST recommendations.

PCAST relies on a large network of subcommittees and working groups that are not subject to the same level of FACA oversight as its principals. These groups typically consist of roughly 10 members from the private sector and are chaired or co-chaired by a PCAST member and an additional one to two more individuals with relevant expertise. Similar to plenary meetings, the subcommittee co-chairs are responsible for scheduling, agenda-setting, and moderating meetings. As these subgroups are made up of nonfederal members, including their co-chairs, the working groups and subcommittees present an opportunity for increased independence from government officials in the early development of policy recommendations.

PCAST staff traditionally consists of one to two executive directors, depending on available funding. This staff is supplemented by policy fellows contracted through the American Association for the Advancement of Science (AAAS) or the Science and Technology Policy Institute (STPI), who contribute to background research and participate in report writing. Report drafts are then revised at the subcommittee or working group level, and finally authorized and “authored” by PCAST principals.

The president is the ostensibly the target audience for formal PCAST reports and letters. However, PCAST has significantly increased the degree to which they engage and make recommendations to both federal and nonfederal stakeholder groups since its founding in 1990 (Somani, 2023). In accordance with FACA rules, PCAST also accepts and publishes all public comments before each plenary meeting and allows public input at open plenary meetings. However, it is unclear exactly how, or to what extent, PCAST is responsive to individual citizens or public stakeholder group input beyond the contributions of working group or subcommittee members. Similar to its legal autonomy, privacy supports PCAST's instrumental role, allowing its members and staff the ability to offer candid feedback during report development.

Chairing PCAST working group and subcommittee activities, as well as taking on a leadership role on the council, requires a significant time commitment, especially in light of PCAST's limited resources and full-time staff. Study chairs need to dedicate tens of person-hours per week to report development and dissemination. By several accounts in the Obama administration, for instance, academic members requested special permission from administrators at their home institutions to reduce their hourly commitments to accommodate their service on PCAST (Gates, 2021; Schaal, 2022). In contrast, during the Clinton and George W. Bush administrations, inactivity of membership due to lack of direct presidential support led to PCAST members recommending that member terms be shortened and that a formal mechanism for stepping down from the council be created (Marburger and Kvamme, 2008; Wu, 2001). Bromley stated that he regretted not tasking PCAST members with more substantive projects, due to both a lack of funding for council operations and the unavailability of members for regular activities in Washington, DC (Bromley, 1994). For PCAST to operate effectively, the council needs to be empowered by the president to commit enough time to its activities and funded at a level that allows for regular interactions between members and relevant stakeholders inside and outside government.

### 4.3 Types of advice

Types of advice refers to PCAST's intended advisory function and how PCAST interprets its mission to offer guidance and policy recommendations on federal STI policy to the president and other public and private stakeholders (Bressers et al., 2018; Craft and Howlett, 2012). While PCAST's intended mission and charter language have evolved with time, it has remained flexible to allow the council to both meet the needs of the administration (solicited advice), as well as offer advice on STI policy issues the council or individual members decide need president-level attention (unsolicited advice). PCAST's advice can be both reactive or anticipatory, address a wide range of audiences, and take various forms depending on its intended audience. PCAST is also responsible for two biennial reports to Congress reviewing the National Nanotechnology Initiative (NNI) and the Networking and Information Technology Research and Development (NITRD) program, discussed below.

This flexibility allows PCAST to provide both short-term, reactive advice, e.g., providing recommendations for building STI capacity at the newly created Department of Homeland Security or informing the federal response to the H1N1 pandemic (PCAST, 2002a, 2009). Its broad mission also allows PCAST the ability to offer anticipatory advice on longstanding policy challenges facing the US national STI ecosystem, e.g., its consistent focus on government-university partnerships (PCAST, 1992b, 1996a, 2004a, 2008, 2012a, 2021), manufacturing and international competitiveness (PCAST, 1992c, 2000, 2002b, 2004b, 2011, 2012b, 2017, 2020, 2022), science, technology, engineering, and mathematics (STEM) education (PCAST, 1992d, 1996b, 1997, 2004a, 2010a, 2012c, 2021), and the role of STI in domestic and national security (PCAST, 1992e, 1995, 2002a, 2003, 2013, 2016a). PCAST reports have become longer and more formalized with time beginning in the Clinton and George W. Bush administrations, mimicking consensus reports of other national advisory bodies, such as NASEM (Somani, 2023). However, PCAST can and often does issue shorter letters or letter reports, especially for items intended to address the president directly (Malcom, 2020). In short, PCAST recommendations, whether published through letters or reports, are intended to be substantive and actionable for policymakers, including the president, senior EOP officials, and representatives in federal STI agencies, and public stakeholder groups. Well-developed policy recommendations that are designed to inform or provide options to decision-makers are a strong signal of PCAST's instrumental advisory role, even if the recommendations are not acted upon (Krick, 2015).

#### 4.3.1 President George H.W. Bush

PCAST's original charter under President George H.W. Bush stated that the council served to “advise the President on matters involving all areas of science and technology,” leaving its intended areas of study up to interpretation by Bromley as sole chair of the council (Executive Office of the President, 1990). PCAST offered a more descriptive mission statement in the front matter of its published reports in 1992:

“Although the boundaries are not clear-cut, the council's advisory work falls broadly into three categories: (1) emerging science and technology issues; (2) policy for science and technology as well as science and technology for policy; and (3) structural and strategic management policies within the Federal government as well as policies in non-governmental organizations” (PCAST, 1992a).

This language mirrors longstanding conceptions of the governance and use of science in policymaking developed by Brooks (1967) that separates the theory and practice of science policy into “science for policy,” e.g., how scientific data and analysis and inform government decision-making and “policy for science,” e.g., the laws, governing principles, regulatory environment, and budgetary priorities that supports the conduct of STI R&D.

In alignment with its stated mission, Bromley organized his PCAST to support the Bush administration's STI policy portfolio, which centered on five cross-cutting initiatives: global change, high performance computing and communication, advanced materials, biotechnology, and science and mathematics education. For each subject area, Bromley assigned two to three PCAST members to review reports from the Federal Coordinating Council for Science (FCCSET), an interagency policy body that served to coordinate federal efforts on the five crosscuts across participating agencies (Bromley, 1992).^10^ FCCSET was created in OSTP's founding legislation in 1976 “to provide more effective planning and administration of federal scientific, engineering, and technological programs” and eliminate duplicative efforts (P. L. 94-282, 1976). FCCSET originally consisted of high-level policy representatives from all STI-related agencies and Cabinet departments with the science advisor serving as chair. However, President Carter, as part of his reorganization of OSTP in 1980, downgraded FCCSET to a sub-Cabinet level committee and moved it out of OSTP, an arrangement that continued under President Reagan (Blanpied, 2010). Bromley fought to revitalize FCCSET, appointing agency heads and Cabinet secretaries as members and encouraging principals to attend meetings, even as some senior White House staff were not enthusiastic of the council's elevated role (Bromley, 1990a).^11^ Bromley's integration of PCAST with FCCSET activities and OSTP policy priorities established a blueprint for the White House STI policymaking and advisory system that remains intact through the present day.

President Bush's PCAST produced seven consensus reports. Recommendations were typically anticipatory rather than reactive, intending to steer federal STI policy toward long-term positive outcomes instead of responding to short-term concerns. The reports were delivered directly to the president through memoranda from Bromley and circulated internally leadership inside the White House. At the end of the administration all seven were published as booklets in December 1992 following the aforementioned lawsuit against OSTP. Intended to be delivered to the president rather than published for review by outside stakeholders, the Bush PCAST reports are relatively short in comparison to later administrations. Out of the seven, only one—the council's final report on STEM education, a lengthy report developed from a series of listening sessions across the US—would be considered a full-length report in the context of more recent PCAST policy products (Hamilton, 1992; PCAST, 1992b).

#### 4.3.2 President William J. Clinton

Under President Clinton, PCAST's mission was similarly broad: “to advise the President…on matters involving science and technology” (Executive Office of the President, 1993a).^12^ President Clinton added one significant function of PCAST in his renewed charter—to advise the National Science and Technology Council (NSTC), a relationship that was not formalized under President Bush (Bromley, 1990b). Launched through executive order the same day as PCAST in 1993 under the leadership of President Clinton' first science advisor, John H. “Jack” Gibbons, NSTC served to update FCCSET in two ways (Executive Office of the President, 1993b). First, it worked to elevate the stature of FCCSET within the White House, which was chaired by the science advisor; NSTC made the president chair. And second, it merged FCCSET with two other science-related White House bodies, the National Space Council and the National Critical Materials Council, in an effort to streamline governance of STI activities.^13^ NSTC has remained active since its creation by President Clinton through the Biden administration. However, in practice, the president has rarely been involved with NSTC activities, despite his position as chair. Instead, the science advisor has remained the *de facto* chair, organizing and managing the committee's working groups and subcommittees (some of which are congressionally mandated) on multi-agency STI programs, and facilitating interaction between PCAST and NSTC.^14^

Similar to President George H.W. Bush, President Clinton's PCAST focused on providing anticipatory advice on longstanding challenges to the national STI enterprise, rather than responding to events or immediate needs of the administration. Especially during President Clinton's first term, its recommendations were published as short-form letter reports that were directly delivered to the president, e.g., PCAST's letters to the president on research universities and the role of federal investment in technology (PCAST, 1996a,b). Notably, President Clinton's PCAST also served as a catalyst for NNI. President Clinton's second science advisor, Neal Lane, tasked PCAST with reviewing the proposed initiative as drafted by the Interagency Working Group on Nanotechnology under NSTC, utilizing the formalized mechanism for PCAST-NSTC interactions. PCAST supported the initiative, recommending that some funding be devoted to studying social and ethical issues surrounding the development of nanotechnology (Lane, 2021; PCAST, 1999; National Research Council, 2002).

During its two terms under President Clinton, PCAST produced 23 publications, 14 of which were letters. This focus on shorter products was an outlier among PCAST's successors, which focused on longer report-length products. Clinton's PCAST publications included detailed reports on federal energy policy on fusion research, federal energy research and development, and international cooperation on energy innovation (Holdren and Baldwin, 2001). Chaired by John Holdren, who later became President Obama's science advisor, these three reports foreshadowed the longer, NASEM-like reports typical of PCAST during subsequent administrations. Lane (2021) later noted that “Holdren was particularly active, at least when I was in the White House,” and “really drove much of the PCAST agenda, consistent with President Clinton's priorities on climate change and renewable energy.” Toward the end of the second term, PCAST also issued two reports on ecological resources and biodiversity led by Peter Raven, which were distinct from other previous or future reports for both their content and funding sources (PCAST, 1998, 2001). Both contained ample color photographs and glossy covers and were funded by the Smithsonian Institution and private sector nonprofit organizations, an exception to PCAST's reliance on government funds to produce study reports.

#### 4.3.3 President George W. Bush

Present George W. Bush ‘s initial charter language for PCAST's advisory role was identical to President Clinton's (Executive Office of the President, 2001). However, after the passage of the 21st Century Nanotechnology Research and Development Act in 2003, which codified NNI into law, President Bush updated PCAST's charter to appoint the council as the National Nanotechnology Advisory Panel (NNAP) (Executive Office of the President, 2004). E. Floyd Kvamme, external co-chair of PCAST, reportedly convinced Senator Ron Wyden (D-OR), the sponsor of that bill, to have PCAST serve in that capacity (Kvamme, 2011). Just over a year later, in 2005, PCAST was merged with the President's Innovation and Technology Advisory Committee (Executive Office of the President, 2005). Similar to PCAST, PITAC was a FACA committed established by the High Performance Computing Act of 1991 to advise on the Networking and Information Technology Research and Development (NITRD) program (P. L. 102-194, 1991). These executive orders instituted a new advisory function for PCAST, one has continued through the Biden administration: to report not just to the president and the White House, but also to Congress.

Led in close collaboration between Kvamme and President Bush's long-serving science advisor, John H. “Jack” Marburger III, PCAST continued its focus on persistent challenges to the US STI system and federal energy policy. Additionally, in the wake of the September 11 attacks in 2001, PCAST produced two early-term studies on the role of STI in domestic and national security—an example of PCAST's potential for reactive advice in service to top priorities of the administration (PCAST, 2002a, 2003). Other report topics were identified through direct interactions with senior administration officials and interviews with Cabinet secretaries. In an exit memorandum to future PCASTs, Marburger and Kvamme (2008) stated that a “close tie between the PCAST and the administration resulted in report topics which were, by and large, tied to current topics of administration interest and, as a result, of use and interest to the administration.”

During Marburger's 7-year tenure as science advisor, PCAST reports continued to evolve to address a wider range of public and private stakeholders and became more consistently formatted (Somani, 2023). President Bush's PCAST produced 18 reports, most of which took the form of longer, more involved consensus reports that were circulated to stakeholders both inside and outside of the executive branch rather than internal letters intended only for the president. In the same exit memorandum, Marburger and Kvamme (2008) recounted that “by early agreement, reports were not lengthy but rather held to a model of having 30–50 pages with recommendations that were immediately actionable as ‘first steps' in moving in a recommended direction” (Webb, 2001). The memorandum also states that PCAST could have benefitted from more frequent engagement with federal representatives, other White House policy councils, or federal advisory bodies, such as Office of Management and Budget, the Council of Economic Advisers, and the DSB, as well as with Congress. Such interaction could have broadened awareness of PCAST activities in support of its policy recommendations.

#### 4.3.4 President Barack Obama

President Obama's charter for PCAST offered a more prescriptive role for the council, including naming priority areas for study in its mission statement. Beyond advising on matters involving STI policy, PCAST's updated charter states that the council's “advice shall include, but not be limited to, policy that affects science, technology, and innovation, as well as scientific and technical information that is needed to inform public policy relating to the economy, energy, environment, public health, national and homeland security, and other topics” (Executive Office of the President, 2010). The Obama administration continued the precedent established by President George W. Bush of PCAST serving as and NNAP and PITAC, requiring the council to produce biennial reports to Congress on NNI and NITRD. Under President Obama, PCAST also adopted the reports and the recommendations of the steering committee to the Advanced Manufacturing Partnership (AMP) and Advanced Manufacturing Partnership 2.0 (AMP2.0) (PCAST, 2011, 2012b).

PCAST's activities to President Obama and his administration stood out from its contemporaries. First, the council was significantly more productive in its 8 years of operation than any other recent PCAST, producing 36 letters or reports and 440 consensus recommendations. Second, PCAST pursued studies that were outside of the council's traditional wheelhouse in government-university partnerships, STI competitiveness, STEM education, and national security, expanding the range of both anticipatory and reactive advice PCAST typically offers. In particular, two PCAST reports—one providing recommendations on improving hearing technologies and one reviewing scientific practices in forensic evidence—have since proved to have clear, lasting impact beyond the end of the Obama administration, leading to policy reforms (PCAST, 2015, 2016b). The former led to a US Food and Drug Administration (FDA) regulatory change to allow for the purchase of over-the-counter hearing aids and the latter has provided the US legal system with new scientific context for the admission of forensic evidence in criminal and civil law cases.

PCAST also produced reports responsive to other key areas of the administration's broader policy agenda on health care informatics, climate change, vaccine policy, and drinking water—more examples of PCAST's ability to provide reactive STI policy advice. The direction for these areas often originated from engagement with President Obama at the start of the administration, who identified a number of key issues PCAST should address during its first meeting from a menu of possible study themes put together by PCAST. Addressing pandemics and expediting vaccine development were the priority areas Obama identified (PCAST, 2009, 2010b; Moniz, 2020). Indeed, the first Obama (PCAST, 2009) report on the H1N1 pandemic led to the creation of the Directorate for Global Health Security and Biodefense under the National Security Council (NSC) in 2015, which coordinated government-wide pandemic preparedness. Holdren later said that PCAST and NSC were “joined at the hip,” which helped facilitate the uptake of PCAST's recommendations on issues related to national security (McLaughlin, 2017). Additionally, the close working relationship between PCAST and President Obama, enabled a strong alignment between the president's policy interests and council activities, likely leading to more direct influence on White House decision-making.

#### 4.3.5 President Donald J. Trump

President Trump slightly altered PCAST's charter to advise on “matters involving science, technology, education, and innovation policy” and to “provide the President with scientific and technical information that is needed to inform public policy relating to the American economy, the American worker, national and homeland security, and other topics” (Executive Office of the President, 2019). This language reflected the Trump administration's broader STI policy agenda, which focused the contributions of STI R&D to the US economy and national security.

President Trump's PCAST, however, suffered from a comparatively late appointment of its chair—science advisor, Kelvin Droegemeier. PCAST members were not appointed until after Droegemeier's confirmation by the Senate over 2 years into the administration. In the remaining 2 years of the administration, PCAST produced three reports: one *pro forma* report reviewing NITRD and two reports on what the Trump administration called “industries of the future” (IoTF), a term that was subsequently picked up and used by the Biden administration (PCAST, 2021). These two reports offered recommendations for strengthening the US STI leadership in five areas: artificial intelligence, quantum information science, advanced manufacturing, biotechnology, and advanced communications networks.

In February 2020, Droegemeier organized the first ever joint meeting between the principals of PCAST and NSB, the policy arm of the National Science Foundation (NSF). Droegemeier had previously served as vice chair for NSB and saw an opportunity for the two bodies to complement each other, especially related to STEM workforce issues (Droegemeier, 2022). Establishing areas of mutual interest during their first meeting, Droegemeier then appointed NSB liaisons to participate in report development of both IoTF reports. NSB plenaries, just like PCAST members, are similarly experienced and eminent scientific professionals that are well-positioned to inform and disseminate PCAST policy reports and recommendations. Active engagement with NSB provided a mechanism to potentially strengthen the substantiveness of PCAST's IoTF reports and expand the range of potential audiences in both the public and private sector.

#### 4.3.6 President Joseph R. Biden

PCAST's intended advisory role under President Biden drew from language of the Obama charter—advising on topics covering both “policy for science” and “science for policy.” The new mission also expanded the council's remit to include other areas of domestic policy, including “the economy, worker empowerment, education, energy, the environment, public health, national and homeland security, racial equity, and other topics” (Executive Office of the President, 2021). The renewed executive order arrived less than 2 weeks after President Biden sent a letter to his first science advisor, Eric Lander, which posed five questions intended to shape the work of OSTP and by extension, PCAST (White House, 2023b). The five topics included: improving pandemic response, addressing climate change, strengthening international competitiveness, increasing the societal returns of STI R&D, and ensuring the long-term health of the STI enterprise.

PCAST's activities early in the administration followed the suggested topics put forth by President Biden's letter. Lander, who had served as an external co-chair of PCAST during the Obama administration and later, Arati Prabhakar, President Biden's second science advisor, continued the council's tradition of publishing substantive, anticipatory consensus reports.^15^ The council's first report, however, was reactive—it provided recommendations for the implementation of the recently passed CHIPS and Science Act (PCAST, 2022; P. L. 117-167, 2022).

### 4.4 Publicity

Publicity refers to the effort paid by the administration toward creating a positive public image of the committee and drawing public attention and support to committee activities (Boswell, 2008; Krick, 2015). Since its creation under President George H.W. Bush, PCAST has served as a mechanism for the president to visibly represent the administration's STI priorities and his commitment to the scientific community through the appointment of distinguished scientists and engineers (Evans and Matthews, 2024). Although the bulk of PCAST operations occur in private, the administration works to garner media attention to the committee's launch, public meetings, and published consensus reports, especially during the presidential transition and early in the administration. The consistent effort to generate favorable visibility for PCAST across presidential administrations signals a strong symbolic advisory role. In a purely instrumental role, the administration would be indifferent to whether the public knows or cares about the advice PCAST provides (Boswell, 2008).

PCAST's publicity and symbolic advisory role traces back to the council's creation by President George H.W. Bush. Bromley was a well-known figure within the STI policy community. Just prior to his appointment as science advisor, he received the National Medal of Science in 1988 and served as president of AAAS, the world's largest scientific professional organization. He had also served on the White House Science Council (WHSC), the immediate predecessor to PCAST under President Reagan and as a member of NSB from 1988 to 1989, rolling off to take the position as science advisor.^16^ Bromley's appointment and his role in the creation of PCAST was lauded by the STI community and received ample positive media attention by national news organizations (Beardsley, 1989; Culliton, 1989; Dowd, 1990; Mervis, 1990). PCAST members were sworn in by Vice President Quayle and the council's first meeting was held at Camp David with the president (Goodwin, 1990; Kremer, 1990). The swearing in ceremony and the Camp David visit received also media attention, including photo opportunities with each PCAST member shaking hands with the vice president and president taken by the White House photographer—a strong indication of the administration's intent to create visibility and a positive public image of the committee (Woodson Research Center, 2024).

The tradition of appointing high-profile science advisors and its favorable reception by the media continued in each administration that followed. President-elect Clinton announced Gibbons, former director of Oak Ridge National Laboratory and long-serving director of the Congressional Office of Technology Assessment, as his science advisor alongside the members of his Cabinet on a nationally televised event (Clinton, 1993; Schrage, 1993). The announcement of Lane's appointment to replace Gibbons was also scheduled to address a large public audience: Clinton announced Lane, who was then in his fifth year of a planned 6-year term as NSF Director, as his new science advisor at the annual meeting of AAAS to audience of scientists, which included his members of his PCAST (Clinton, 1998). President George W. Bush announced Kvamme as PCAST co-chair at a nationally televised press briefing on his economic policy, which included CEOs of major technology corporations and several members of his Cabinet (Bush, 2001). The subsequent appointments of Marburger, former director of Brookhaven National Laboratory and president of Stony Brook University, and his PCAST also received positive media coverage (Allen, 2001; Gugliotta, 2001; Pear, 2001; Webb, 2001). President elect Obama, similar to Clinton, announced his science team—including Holdren as science advisor and PCAST co-chairs Harold Varmus and Eric Lander—at a C-SPAN covered press release (Obama, 2008).

PCAST under President Trump, received comparatively less media attention than its contemporaries, likely due, in part, by the council's late appointment and the frequent criticism from media outlets regarding President Trump's treatment of science early and throughout his presidency (Guarino, 2019; Mervis, 2019). Nevertheless, Droegemeier—a distinguished meteorologist who had recently served as vice chair of NSB—stressed the importance and value of PCAST to the administration in early press engagement (Droegemeier, 2019a,b). President Biden's rollout for PCAST included both a preinaugural announcement of PCAST's co-chairs and a recorded two-and-a-half-minute video on YouTube of the president lauding the council's preeminent membership and its importance to his administration, which were shared on social media (Biden, 2021; Biden-Harris Transition, 2021).

Both the public statements from the presidents and ensuing media coverage intended to create an image of strong scientific leadership within the White House, as well as close relationships to leading figures in academic science, private industry, and medicine. While PCAST media mentions typically waned after the council's initial appointments, PCASTs have made sustained efforts to draw attention to council's activities, inviting press to plenary meetings, holding public briefings on the release of policy reports, and, in more recent administrations, publishing blog posts and promoting engagement on social media. PCAST therefore carries a strong symbolic role, serving as a visible representation of the administration's commitment to STI, regardless of how administration engages with the council after its initial appointment.

## 5 Discussion

PCAST's broad mission, limited statutory requirements, and the flexibility of FACA allow presidents and their science advisors significant freedom in how they choose to organize and manage their administration's PCAST. Despite differences in its governance and utilization across presidencies, each president's PCAST has carried both a strong instrumental advisory role, as evidenced by the opacity of most member interactions and the substantiveness of its policy recommendations, and a symbolic advisory role, consistent with the sustained efforts of the White House to craft a favorable public image of the council.

PCAST's proximity to the president, the role of the science advisor as chair or co-chair, and the council's ability to “socialize” its recommendations, highlight the council's “contested autonomy” from government control. As former PCAST member Ernest Moniz (2020) describes, PCAST is “sort of on the inside and on the outside [of government] at the same time.” PCAST's legal and economic autonomy have remained consistent since its founding in 1990: the council is governed by FACA and is funded, with rare exceptions, by the government. However, each president and their science advisors have approached PCAST's organizational autonomy with different strategies to either align the council's activities with administration priorities or offer it more independence. PCAST can simultaneously operate in public as an extension of the White House policymaking apparatus, and in private as confidential group of advisors to the president. Future PCASTs should look to President Obama's PCAST as a model for navigating FACA's transparency requirements as a means to ensuring candid negotiations and conflict resolution, especially when counseling the president directly. During two administrations—Presidents George H.W. Bush and Trump—the science advisor served as the sole chair, rather than co-chair. This arrangement suggests stronger government control of council activities, which was reflected in the comparatively limited range of topics of their policy activities relative to other recent PCASTs. To promote council independence and expand the utility of PCAST's advice beyond administration priorities, PCAST should appoint at least one external co-chair to hold the same level of authority over council decision-making as the science advisor.

Decision-making conditions for PCAST have shifted across presidencies. In most recent administrations, PCAST has suffered from limited budgets, hampering its ability to host regular, plenary meeting that facilitate direct involvement with senior White House leadership and encourage members to dedicate time to council work (Shapiro, 2021). President Obama's PCAST was also a notable exception in this regard—its above average annual budgets permitted bimonthly plenary meetings, which the president often attended (Moniz, 2020; Press, 2022, 2023; Savitz, 2021). These meetings and direct presidential involvement appear to have created a culture of responsibility and commitment to service that contributed to the council's high productivity. PCAST needs to be adequately funded to allow for the scheduling of regular plenary meetings and to provide sufficient resources for PCAST studies to avoid individual members self-funding report costs. Additionally, PCAST's leadership structure and balance of represented perspectives changed over time based on the needs of the administration and its STI policy priorities. A high degree of diversity in represented social and professional perspectives can increase the epistemic quality of PCAST's consensus recommendations, which strengthens the council's instrumental advisory role (Bohman, 2000; Brown, 2009). The Biden administration's efforts to increase the participation of women and minoritized and historically marginalized racial and ethnic groups should be continued (Evans and Matthews, 2024). Additionally, Droegemeier's creation of a PCAST subcommittee of students, postdoctoral scholars, and early career professionals (SPEC) during the Trump administration should be considered by future administrations to increase represented perspectives from junior researchers in council activities.

PCAST advice delivered through its published consensus reports was typically anticipatory rather than reactive, focusing on long-standing challenges in federal STI policy. Most PCAST reports addressed topics related to “policy for science,” rather “science for policy.” Council activities had a consistent focus on government-university partnerships, manufacturing policy and international competitiveness, STEM education, and national security. However, PCAST has, especially when called upon by the president, offered short-term, reactive advice in response to urgent concerns of the administration or pressing issues requiring presidential action. Examples include the early reports on homeland security during the George W. Bush administration and PCAST's first report under both President Obama and President Biden on the response to the H1N1 virus and the implementation of the recently passed CHIPS Act, respectively. While PCAST's intended audience for its advice was ostensibly the president, PCAST consensus reports and its policy recommendations address many different stakeholders, both inside and outside government (Somani, 2023). In each administration, regardless of the council's independence and decision-making conditions, PCAST intended to provide substantive, actionable recommendations, signaling a strong instrumental advisory role. PCAST should continue its tradition of choosing study topics that will be of interest to the president and the administration, as well as pursue studies that the council believes require presidential level attention. To maximize impact of this advice, PCAST can tailor its products to its audience, either writing lengthy NASEM-like policy reports or delivering letters and memoranda directly to senior officials, including the president. In response to its statutory obligations as NNAP and PITAC, PCAST can issue letter reports—shorter consensus documents that fulfill its requirements as those two bodies, but do not require the same resources or time commitments as full-length studies.

PCAST's advisory role has also been shaped by each administration's efforts to present the council as an influential and trusted resource on STI policy for the administration, despite limited involvement by the president and inconsistent engagement with senior White House officials beyond the science advisor. Public announcements of PCAST appointments focused on the group's prestige and eminence, which has consistently included visible members from the academic research community, senior executives of major technology companies, and former high-level government officials. Media coverage of PCAST from scientific journals and national news organizations has been overwhelmingly favorable. These efforts signal that beyond PCAST's core instrumental role to provide policy advice to policymakers, the council also carries a strong symbolic role, serving as a vehicle for communicating to the public, especially the STEM community, the importance of STI to the president and their administration. Future administrations should continue to promote PCAST's work and highlight its importance to the administration through active engagement with news organizations and senior White House officials, including the president. Regular and direct involvement from the president supports both advisory functions: it ensures PCAST activities are aligned with the administration's policy priorities, and it legitimizes and draws attention to the council and the advice it provides. This visibility facilitates the broad dissemination, awareness, and potential uptake of PCAST policy recommendations among stakeholder groups inside and outside of government.

## 6 Conclusion

This paper presents the first comprehensive assessment of PCAST's organization, operations, and influence from 1990 to 2023. We build on existing analytical frameworks for evaluating the function and autonomy of expert bodies to explore PCAST's instrumental and symbolic advisory roles across time and presidential administrations. We find that PCAST has engaged with both advisory roles in all recent presidencies. However, we demonstrate that its independence from government control, its decision-making conditions, and the nature and intended audience of its policy advice has been variable. The paper concludes with recommendations for organizing and managing future PCASTs to ensure they are well-positioned to appropriately influence presidential level decision-making.

This paper contributes to existing literature on national STI advisory bodies as boundary organizations, providing the first detailed examination of PCAST's operational history. Our findings offer new data, analysis, and historical context for future studies examining the role of science and scientists in shaping US national STI policy. More research is needed to understand how and when PCAST recommendations have translated into tangible policy outcomes, such as presidential budget requests to Congress, executive orders, presidential decision memoranda, regulations, legislation, or other statements of policy. Developing a measure of expert influence in government decision-making would provide a means to better understand the changing nature of scientific authority in federal policymaking in the United States.

## Data Availability

The data generated in this study are available in public repositories of Woodson Research Center (2024) and Science History Institute (2024).

## Footnotes

^1^Policy recommendations for improving the overall US national STI R&D enterprise have five broad outcomes: to accelerate the rate of scientific discovery, increase the societal returns of federally-funded R&D, create a more inclusive scientific workforce, and improve international competitiveness, and advance national security.

^2^We use “committee” and “council” interchangeably throughout the paper.

^3^President Eisenhower appointed James R. Killian Jr. as special assistant to the president for science and technology in 1957 (Killian, 1993). Most science advisors have held both titles of APST and OSTP Director: D. Allan Bromley under President George H.W. Bush; John H. Gibbons and Neal F. Lane under President Clinton; John P. Holdren under President Obama; and Eric Lander and Arati Prabhakar under President Biden. President George W. Bush's science advisor John H. Marburger III and President Trump's science advisor Kelvin Droegemeier held just the title of OSTP Director. Francis Collins, in his interim appointment as science advisor under President Biden, was appointed solely as APST. It remains a subject of debate whether a single individual should hold both titles given the inherent tension between executive privilege for APSTs and congressional oversight of OSTP Directors.

^4^While Bromley held both titles of APST and OSTP Director, he served as PCAST chairman in his capacity as OSTP Director. Marburger and Droegemeier, who did not hold the title of APST, also served on PCAST in their capacity as OSTP Director. In Marburger's case, the executive order was issued prior to his appointment, so it reads the co-chair will be a “federal government official designated by the president,” rather than OSTP Director. All other science advisors—Gibbons, Lane, Holdren, Lander, and Prabhakar—served as PCAST co-chairs in their capacity as APST.

^5^PCAST's leadership structure has changed across presidential administrations. President George H.W. Bush appointed Bromley as a sole chairman with a nonfederal member as vice chairman. President Trump appointed Droegemeier as the sole chair without a vice chair. President's Clinton and George W. Bush each had two co-chairs—one federal, one nonfederal. Presidents Obama and Biden allowed for three co-chairs: one federal, two nonfederal. President Obama's PCAST also saw the appointment of two vice chairs after one nonfederal co-chair (Harold Varmus) rolled off of the committee.

^6^President Clinton's initial lack of participation likely stemmed, in part, from his delegation of STI policy to Vice President Gore (Broad, 1992). Further, Vice President Gore had a longstanding relationship to President Clinton's first science advisor, John H. Gibbons, with whom he had developed a close working relationship with during his tenure in Congress; Gibbons served as both director of Oak Ridge National Laboratory in Tennessee and later director of the US Congress Office of Technology Assessment (OTA). Moniz (2020) suggested the Vice President Gore was responsible for Gibbons' appointment due to the Tennessee connection and his visible interest in STI policy.

^7^Wells, Jr. also recounted that “Darman kept a notebook on alleged OSTP and Bromley transgressions and that Darman and [Chairman of the Council of Economic Advisers Michael J.] Boskin ‘took Allan to the woodshed' to berate him for technology policy activism and other matters of supposedly talking out of turn.” He concluded by stating that “only [Bromley's] regard for the president kept [the situation] under wraps” (Wells, 1993).

^8^Walter Massey and Bernadine Healy rolled off roughly a year into their service on President George H.W. Bush's PCAST to serve as the Directors of the National Science Foundation (NSF) and the National Institutes of Health (NIH), respectively. Similarly, Harold Varmus during the Obama administration left to serve as NIH Director. Dario Gil rotated off President Trump's PCAST to serve on NSF's National Science Board and Shirley Ann Jackson left to chair the President's Intelligence Advisory Board to avoid appearing to be able to “advise themselves” by serving on both committees at once. Other departures included Phillip Sharp during the Clinton administration, Stephen Burk and Gordon Moore during the George W. Bush administration, Richard Levin during the Obama administration, and Penny Pritzker during the Biden administration. Jack Gibbons left as science advisor and co-chair of PCAST under President Clinton during his second term, having made it clear that he only intended to serve one term (Bierbaum, 2021; Wells, 1996). Ashton Carter passed away in October 2022 during his tenure on President's Bident's PCAST.

^9^President Bush's industry-heavy PCAST resulted, in part, from its merger with the President's Innovation and Technology Advisory Committee (PITAC) in 2005. To take on PITAC's advisory responsibilities, PCAST's charter was revised to expand maximum membership to forty-five. The following year, in 2006, President Bush appointed fourteen new members with backgrounds in computing, telecommunications, and information systems, which tilted PCAST's membership majority to industry perspectives. Only one new members, Daniel Reed, had served on PITAC before the council was dissolved.

^10^FCCSET is pronounced “fix-it.”

^11^President Bush's chief of staff John Sununu wrote to White House staff secretary Jim Cicconi in September 1990 shortly after FCCSET's restructuring that the council “raises very, very serious questions about the organization of the White House, the position of the [Economic Policy Council] and [Domestic Policy Council], and the way in which policy decisions are taken here. Frankly, since FCCSET is the only domestic policy group in the [Executive Office of the President] established by law, it also raises potential questions of Congressional power over the White House policy processes” (Bromley, 1990a).

^12^President Clinton also changed the “C” in PCAST from “council” to “committee.” President George W. Bush changed it back to “council,” which has continued through the first term of the Biden administration.

^13^Despite Bromley's efforts to revitalize FCCSET, the Clinton administration decided to overhaul the council as part of its National Performance Review initiative to streamline government activities. The National Performance Review report, chaired by Vice President Gore, stated that “Unfortunately, FCCSET lacks the teeth to set priorities, direct policy, and participate fully in the budget process. It can't compel agencies to participate in its projects, nor can it tell agencies how to spend funds” (National Performance Review, 1993). It continued in support of NSTC by stating that, “A new National Science and Technology Council would direct science and technology policy more forcefully, and would streamline the White House's advisory apparatus by combining the functions of FCCSET, the National Space Council, and the National Critical Materials Council.”

^14^Internal to White House operations, NSTC is challenging to tract from the public record outside of periodic reports from the Congressional Research Service (Blevins, 2023). For a discussion on NSTC and strategies for interagency STI collaboration see Blackburn (2016).

^15^Lander resigned as science advisor in February 2022 after reporting and an internal investigation uncovered that he violated workplace conduct rules, bullying demeaning members of his staff, especially women (Seide and Gold, 2022; Thompson, 2022).

^16^WHSC is pronounced “whisk.”
